# Positronics of subnanometer atomistic imperfections in solids as a high-informative structure characterization tool

**DOI:** 10.1186/s11671-015-0764-z

**Published:** 2015-02-19

**Authors:** Oleh Shpotyuk, Jacek Filipecki, Adam Ingram, Roman Golovchak, Mykola Vakiv, Halyna Klym, Valentyna Balitska, Mykhaylo Shpotyuk, Andrzej Kozdras

**Affiliations:** Lviv Institute of Materials of SRC “Carat”, Stryjska str., 202, Lviv, 79031 Ukraine; Jan Dlugosz University, Al. Armii Krajowej, 13/15, Czestochowa, 42201 Poland; Opole University of Technology, Ozimska str., 75, Opole, 45370 Poland; Lviv Polytechnic National University, Bandera str., 12, Lviv, 79013 Ukraine; Lviv State University of Vital Activity Safety, Kleparivska str., 35, Lviv, 79007 Ukraine

**Keywords:** Nanomaterials, Subatomic void, Positron annihilation lifetime, Positronics, Trapping

## Abstract

Methodological possibilities of positron annihilation lifetime (PAL) spectroscopy applied to characterize different types of nanomaterials treated within three-term fitting procedure are critically reconsidered. In contrast to conventional three-term analysis based on admixed positron- and positronium-trapping modes, the process of nanostructurization is considered as substitutional positron-positronium trapping within the same host matrix. Developed formalism allows estimate interfacial void volumes responsible for positron trapping and characteristic bulk positron lifetimes in nanoparticle-affected inhomogeneous media. This algorithm was well justified at the example of thermally induced nanostructurization occurring in 80GeSe_2_-20Ga_2_Se_3_ glass.

## Background

Progress in the modern nanomaterial science relies, to a great extent, on high-informative characterization probes sensitive to length scales of atomic and subatomic ranges. One of such probes is grounded on positronics, e.g., analytical space-time continuum determination for electron interaction with its antiparticle, the positron.

This phenomenon realized as positron annihilation lifetime (PAL) spectroscopy can be applied to study atomistic imperfections such as free-volume defects (vacancies, vacancy-like clusters, voids and void agglomerates, pores and even macroscopic cracks, etc.) in different solids despite their structural organization (crystals or glasses, fine-grained or coarse powders, ceramics or bulk alloys) [1-3]. In fact, such imperfections evolve subnanometer (angstrom-scaled) low-electron-density spaces stretching far below characteristic sizes of a few bond lengths, a level which is beyond measuring possibilities for many direct experimental structural probes (such as optical or electron microscopy). In application to semiconductors, this method allows useful identification of intrinsic-free volumes owing to simple models considering competitive channels of positron trapping from delocalized defect-free bulk states, deep ground states of positron traps (extended free-volume defects), and pick-off decaying of bounded positron-electron (positronium Ps) states [1,2]. But when dealing with nanomaterials possessing nanostructural inhomogeneities, the PAL method seems too ambiguous in view of numerous complications in the adequate meaningful interpretation of the detected PAL spectra.

In this work, we shall analyze the possibilities of PAL technique (the positronics) to characterize free-volume void evolution processes caused by nanostructurization in nanoparticle-embedded substances, where intrinsic inclusions can affect both positron- and Ps-trapping channels in the overall balance of annihilation events possible in a host matrix.

## Methods

It is well known that in a majority of nanostructurized systems, the PAL spectra typically yield three distinct lifetime components *τ*_1_, *τ*_2_, and *τ*_3_ with *I*_1_, *I*_2_, and *I*_3_ relative intensities (*I*_1_ + *I*_2_ + *I*_3_ = 1) reflecting competitive input from positron and Ps-trapping channels [1,2].

The longest PAL component (*τ*_3_, *I*_3_) originates from the decaying of spin-triplet ortho-positronium o-Ps states in free-volume holes of the material. This process stretched intrinsically in a vacuum ending by emitting three γ-rays with theoretical lifetime of 142 ns, whereas in a matter, it is quenched in shorten lifetimes of a few nanoseconds because of Ps interaction with electrons from a surrounding medium. As a result, the o-Ps annihilates extrinsically in a medium-emitting two γ-rays, the process known as pick-off annihilation [1,2]. A simple empirical relation in the form of

1 $$ {\tau}_{\mathbf{3}}=0.5\cdot {\left[1-\frac{R}{R+\varDelta R}+\frac{1}{2\pi}\cdot sin\left(\frac{2\pi R}{R+\varDelta R}\right)\right]}^{-1} $$

with material-related parameter *ΔR* equivalent to electron layer thickness for Ps penetration wall region of free-volume trapping void (1.66 Å) was proposed to account for the o-Ps lifetimes *τ*_3_ in dependence on void radii *R* in molecular substrates such as polymers, using infinite potential spherical model initially developed by S.J. Tao [4].

The intermediate PAL component (*τ*_2_, *I*_2_) results from positron (not Ps) annihilation from free-volume defect sites such as atomic and subatomic voids (vacancies, vacancy-like clusters, etc.). Being trapped by such defect, the positron annihilate with character lifetimes *τ*_2_ = *τ*_d_ ranging from 0.2-0.3 to 0.5 ns [1]. This channel of positron annihilation is interbalanced by input from delocalized positrons annihilating directly from defect-free bulk states, thus forming a character two-state trapping scheme with compensating (*τ*_1_, *I*_1_) component and average positron lifetime *τ*_av_ defined as [5]

2 $$ {\tau}_{\mathbf{av}}={\eta}_b\cdot {\tau}_b+{\eta}_d\cdot {\tau}_d={\tau}_1\cdot {I}_1+{\tau}_2\cdot {I}_2, $$

where *η*_*d*_ and *η*_*b*_ is fraction of defect-trapped and free-annihilated positrons, respectively (*η*_*d*_ = 1 − *η*_*b*_). Thus, the shorter *τ*_1_ component is only the reduced bulk lifetime, which occurs to be directly connected with defect-free bulk positron lifetime *τ*_b_ as

3 $$ {\tau}_b=\frac{\tau_1{\tau}_2}{I_1{\tau}_2+{I}_2{\tau}_1}. $$

In case of three-component fitting of PAL spectra, this situation is disturbed by additional input in the first (*τ*_1_, *I*_1_) component from spin-singlet para-positronium p-Ps states, giving *τ*_*p*_ = 0.125 ns with relative population *I*_*p*_ = *I*_3_*/*3 for vacuum. So in reality, the annihilation from defect-free bulk states are admixed to p-Ps decaying channel, making essential complication in a physical meaning of this component. That is why the direct correlations are not allowed often for this (*τ*_1_, *I*_1_) component and material-related parameters of the studied media [6,7].

In our model, we shall try to distinguish these inputs in the first PAL component to release “pure” feedback caused by nanosized free-volume positron traps themselves. Hence, by assuming an additive two-state positron-trapping model for these defect states, we obtain a realistic possibility of their quantitative parameterization. Like [8,9], we use the generalization procedure allowing transformation of the measured PAL spectra from three-term to two-term decomposition form.

Firstly, this analysis has to be applied to host matrix without embedded nanoparticles having (*τ*_1_^host^, *I*_1_^host^), (*τ*_2_^host^, *I*_2_^host^), and (*τ*_3_^host^, *I*_3_^host^) component inputs in three-term decomposed row PAL spectrum (*I*_1_^host^ + *I*_2_^host^ + *I*_3_^host^ = 1). This model can be easily transferred to two-term trapping one by removing *I*_*p*_ = *I*_3_*/*3 input of p-Ps annihilation with *τ*_*p*_ = 0.125 ns lifetime from the first channel and (*τ*_3_^host^, *I*_3_^host^) input from the third channel to the generalized trapping channel. Thus, we can estimate the contribution (*τ*_*a*_, *I*_*a*_) to the first channel other than p-Ps:

4 $$ {\tau}_a\cdot {I}_a = {\tau_{\mathbf{1}}}^{host}{\tau_1}^{\mathbf{host}}\cdot {I_1}^{host}{I_{\mathbf{1}}}^{\mathbf{host}}\hbox{--} {\tau}_p\cdot {I}_p; $$

5 $$ {I}_a = {I_{\mathbf{1}}}^{\boldsymbol{h}ost}{I_{\mathbf{1}}}^{\mathbf{host}}\hbox{--} {I}_p. $$

Then, returning to nanoparticle-embedded sample having (*τ*_1_^*^, *I*_1_^*^), (*τ*_2_^*^, *I*_2_^*^), and (*τ*_3_^*^, *I*_3_^*^) inputs in three-term decomposed PAL spectrum (*I*_1_^*^ + *I*_2_^*^ + *I*_3_^*^ = 1), we can find input from additional trapping channel (*τ*_int_, *I*_int_) assuming that the second channel is composed by these *int*-sites and remainder of *o-Ps*-trapping sites taken like as in a host matrix, so that

6 $$ {I_2}^{*} = {I}_{int} + {I_3}^{*}{I}_{\mathbf{int}}+{I_3}^{*}\cdot \left({I_2}^{host}/{I_3}^{host}\right)\left({I_{\mathbf{2}}}^{\mathbf{host}}/{I_{\mathbf{3}}}^{\mathbf{host}}\right), $$

7 $$ {\tau_2}^{*}\cdot {I_2}^{*} = {\tau}_{int}{\tau}_{\mathbf{int}}\cdot {I}_{int}{I}_{\mathbf{int}} + {\tau_2}^{host}{\tau_2}^{\mathbf{host}}\cdot \left({I_2}^{*}-{I}_{int}\right)\left({I_2}^{*}-{I}_{\mathbf{int}}\right). $$

By transferring three-term PAL spectrum into two-term one with (*τ*_a_^*^, *I*_a_^*^) and (*τ*_t_^*^, *I*_t_^*^) components like in host matrix, we can find compensating (*τ*_*n*_, *I*_*n*_) input arising from this additional trapping channel (*τ*_int_, *I*_int_). Under this condition, it seems quite reasonably to equilibrate the (*τ*_*n*_ · *I*_*n*_) input in (*τ*_a_^*^, *I*_a_^*^) component (without p-Ps) with the same as the (*τ*_int_ · *I*_int_) input in (*τ*_2_^*^, *I*_2_^*^):

8 $$ {\tau}_n\cdot \frac{I_n}{{\tau_a}^{*}}\cdot {I_a}^{*} = {\tau}_{int}{\tau}_{\mathbf{int}}\cdot \frac{I_{int}{I}_{\mathbf{int}}}{{\tau_2}^{*}}\cdot {I_{\mathbf{2}}}^{*}{I_2}^{*}. $$

Physical parameterization of nanoparticle-related sites can be finally performed by accepting the (*τ*_*n*_, *I*_*n*_) and (*τ*_int_, *I*_int_) inputs as corresponding components of the generalized two-term decomposed PAL spectrum of substance affected by embedded nanoparticles. The second component with defect-related *τ*_int_ lifetime reflects the positron-trapping sites appearing due to embedded nanoparticles themselves. Under accepted prerequisites, these extended free-volume defects can be associated with pseudogap holes at the interface between the outer surface layer of agglomerated nanoparticles and innermost layer of surrounding host matrix (as it was well outlined in [8,9]). The bulk positron lifetime recalculated respectively to these components in full agreement to Equation 3 can be attributed to bulk positron lifetime of agglomerated nanoparticles. In case of highly monolith particles, this value tends towards bulk positron lifetime of corresponding substance, while in more loose media, it remains higher. The positron-trapping rate of nanoparticle-related traps *κ*_*d*_ can be also estimated in terms of known two-state positron-trapping formalism [1-3].

Thus, the developed approach allows description of nanostructurization in terms of *substitutional positron-Ps trapping* within the same host matrix, e.g., the process, which occurs as a transformation of o-Ps-sites in a host matrix towards positron-trapping sites in a nanoparticle-modified material. By accepting a tightly connected nature of these PAL trapping sites, we can define conditionally this approach as *coupling x*3*-x*2*-decomposition algorithm* to distinguish it from *conventional x*3*-decomposition procedure*, describing the PAL spectra in terms of admixed positron-Ps trapping. Under such transformation, the quantitative characteristics of these trapping sites themselves as well as the occurring final balance in the PAL components are not so important. However, if the process of nanostructurization is stretched in principally the other way, by example, as modification in defect-free annihilation channel changing essentially PAL parameters describing positron annihilation from delocalized Bloch states throughout a whole host matrix, this algorithm cannot be further applied (new channels of positron annihilation appear in the final three-term decomposed PAL spectrum, which are not foreseen within this simplification procedure). So, this algorithm is expected to serve like *test indicator* for nanostructurization nature in different types of materials, separating the processes of host matrix modification from “pure” interplay between positron- and o-Ps-trapping channels.

## Results and discussion

As an example, let us consider free-volume void evolution in 80GeSe_2_-20Ga_2_Se_3_ glasses caused by thermal annealing at 380°C for 10, 25, and 50 h [10,11]. This thermal treatment is essential for controlled ceramization causing a possibility to stabilize constituting amounts of some nanocrystals (Ga_2_Se_3_, GeSe_2_ and/or GeGa_4_Se_8_) in the final structure of reproducible glass ceramics. The fundamental role of nanosized domains appear by phase separation between Ge- and Ga-rich regions. Higher stability of such thermally treated system is ensured due to its modified structure with some nanocrystallites; this process being arranged in two tightly interconnected stages [11-13]. Firstly, the nucleation of growing crystal phase in glassy environment occurs at the beginning of thermal annealing. Further, the nucleated phase grows into separate grains reaching greater sizes, but, in general, they do not exceed a characteristic nanosized level (a controllable crystal growth). In respect to two-term decomposed PAL spectra [10], the appeared nanocrystallites modify a free-volume structure of the glass leading to specific fragmentation of larger free-volume entities (positron-trapping sites) into greater number of smaller ones with preliminary void nucleation at the initial stage of annealing. So, this process is not elementary, being composed of two principally different types of free-volume void evolution, the initial agglomeration followed by void fragmentation at the final stages. The sizes of corresponding nanocrystalline inclusions estimated from most characteristic peak observed in the XRD patterns of thermally annealed samples in respect to known Debye-Scherrer equation testifies that they reach near 9 to 10 nm.

**Figure 1:**
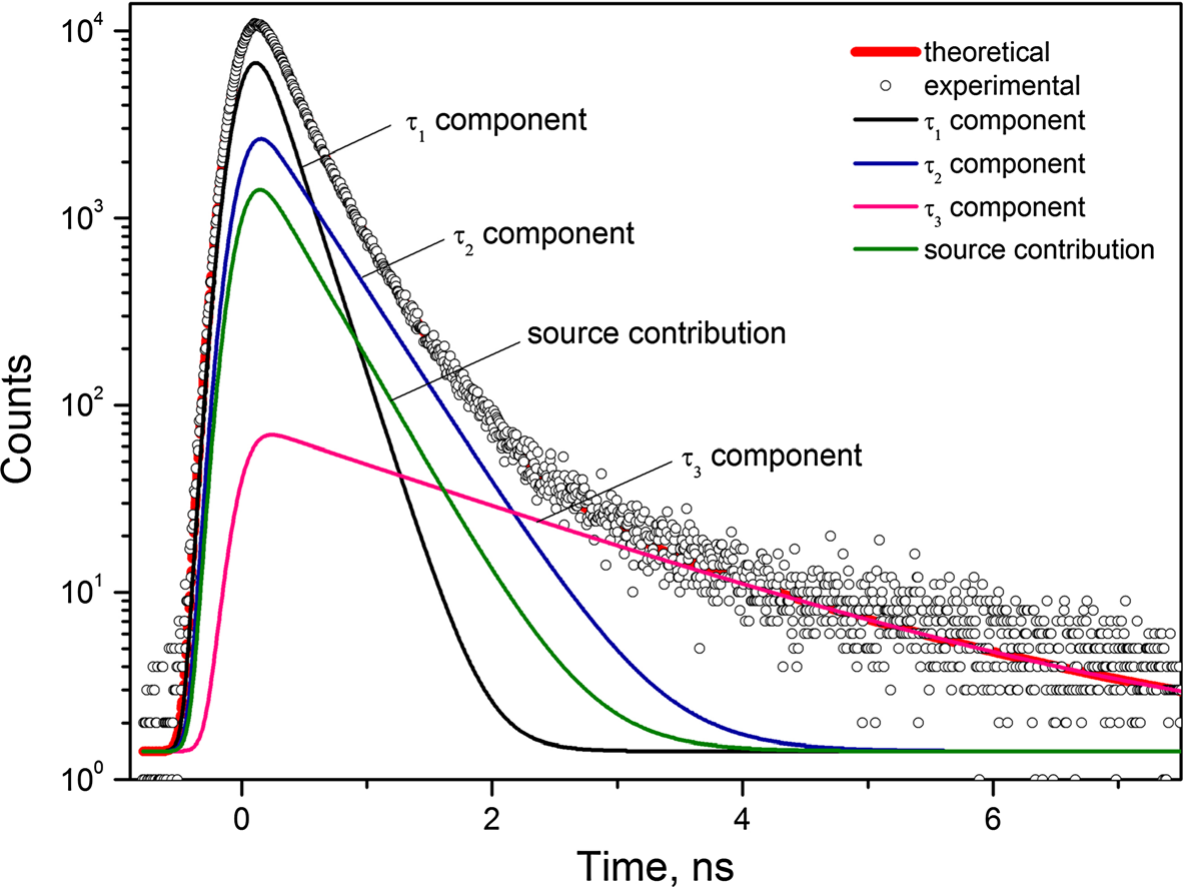
**Raw PAL spectrum of 80GeSe**_**2**_ **-20Ga**_**2**_ **Se**_**3**_ **glass.** Reconstructed from three-term fitting procedure at the general background of standard source contribution.

Typical raw PAL spectrum of initial 80GeSe_2_-20Ga_2_Se_3_ glass (not affected by thermal treatment) reconstructed from three-term fitting procedure at the general background of standard source contribution is shown in Figure 1 (the similar spectra were obtained for all other samples). This PAL spectrum as a typical histogram of elementary positron annihilation events is characterized by a narrow peak and region of long fluent decaying of coincidence counts in a time. Thus, the decaying behavior of such curve is represented by the sum of exponents with different time constants inversed to positron lifetimes [1-3]. The best-fit positron-trapping parameters of the studied samples calculated within three-term fitting are given in Table 1.

**Table 1:** **Fitting parameters and corresponding trapping modes**

**Sample, prehistory**	**Fitting parameters**						**PAL trapping modes**		
	***τ***_**1**_ **ns**	***I***_**1**_ **a.u.**	***τ***_**2**_ **ns**	***I***_**2**_ **a.u.**	***τ***_**3**_ **ns**	***I***_**3**_ **a.u.**	***τ***_**av**_ **ns**	***τ***_**b**_ **ns**	***κ***_***d***_ **ns** ^**−1**^
Host glass	0.203	0.527	0.401	0.443	1.892	0.030	0.293	0.262	1.11
Annealed, 10 h	0.214	0.618	0.428	0.351	2.059	0.031	0.291	0.261	0.85
Annealed, 25 h	0.215	0.633	0.432	0.337	2.038	0.030	0.291	0.261	0.81
Annealed, 50 h	0.210	0.605	0.424	0.365	2.159	0.030	0.290	0.259	0.90

These are determined within conventional x3-decomposition procedure for PAL spectra of host and thermally aged 80GeSe2-20Ga2Se3 glasses.

Therefore, to apply the above coupling x3-x2-decomposition algorithm, the PAL spectra of host and thermally annealed 80GeSe_2_-20Ga_2_Se_3_ glasses were recalculated in terms of conventional three-component x3 fitting. It is obvious that third component related to o-Ps decaying plays no essential role in overall nanostructurization, being at the level of statistical deviation (*I*_3_ ≅ 0.031 ns), while *τ*_3_ lifetime slightly grows in all partially crystallized samples. In contrast, the second lifetime component *τ*_2_ reveals an increase and simultaneous *I*_2_ decrease in the initial stages of annealing (10 h) because of preliminary void expansion (void agglomeration) and further decrease in *τ*_2_ lifetimes with simultaneous *I*_2_ increase during more prolonged annealing (25 h and especially 50 h) testifying in a favor of increased number of smaller free volumes (void fragmentation).

In conventional mixed positron- and o-Ps-trapping modes, all samples demonstrate nearly the same average *τ*_av*.*_ and bulk *τ*_b_ positron lifetimes (0.291 and 0.260 ns, respectively). Such great value of bulk positron lifetime *τ*_b_ testify in a favor of rather loose packing of corresponding nanoparticles (bulk crystallites), which is caused by principal impossibility to distinguish inputs from different positron- and o-Ps-trapping modes.

The above coupling x3-x2-decomposition formalism allows this, since “pure” positron-trapping modes alone can be simply extracted at the general background of overall trapping processes as it is well demonstrated in Table 2 in respect to host and 10-h annealed 80GeSe_2_-20Ga_2_Se_3_ glasses.

**Table 2:** **PAL trapping modes for 80GeSe**_**2**_ **-20Ga**_**2**_ **Se**_**3**_ **glasses**

**Sample, prehistory**	***τ***_***n***_ **ns**	***τ***_**int**_ **ns**	***I***_**int**_ **a.u.**	***τ***_**b**_ **ns**	***κ***_***d***_ **ns** ^**−1**^	***τ***_***n***_ **ns**	***τ***_**int**_ **ns**	***I***_**int**_ **a.u.**	***τ***_**b**_ **ns**	***κ***_***d***_ **ns** ^**−1**^
Host glass	-	-	-	-	-	0.160	0.298	0.092	0.205	1.37
Annealed, 10 h	0.160	0.298	−0.092	0.194	1.10	-	-	-	-	-
Annealed, 25 h	0.181	0.302	−0.106	0.197	1.04	0.189	0.332	−0.014	0.226	0.87
Annealed, 50 h	0.158	0.269	−0.064	0.191	1.09	0.163	0.370	0.025	0.203	1.20

These are treated with coupling x3-x2-decomposition algorithm (in respect to host and 10-h-annealed glasses).

The constancy in the intensity of third component (*I*_3_ in Table 1) testifies that positron-trapping sites appear in addition to o-Ps ones, so they are being formed in the interstitial positions between them. But negative values of PAL component *I*_int*.*_ in the reconstructed final x2-spectrum in respect to host glass indicate that realistic process is connected rather with void disappearing, which can be treated as case of void agglomeration. Indeed, under the condition that *I*_3_^*^ ≅ *I*_3_^host^, the decreasing *I*_2_^*^ intensities in Equation 6 provide negative values for *I*_int_. Thus, in full agreement with previous conclusion extracted from x2-decomposed PAL spectra [10], the *I*_int*.*_ intensities attain firstly negative values, this process being quickly saturated in a 50-h annealed sample.

If we reconsider these results in respect to a 10-h annealed sample (the second line in Table 2), we can observe two principally different tendencies in void evolution. The glass annealed for 25 h is still affected by slight void agglomeration (*I*_int*.*_ = −0.014), while host and 50-h annealed glasses are under obvious void fragmentation (appearance of new positron-trapping sites). The bulk positron lifetimes in all samples are smaller than previously observed *τ*_b_ ≅ 0.260 ns [10], corresponding to positron trapping occurring just in the nanocrystalline particles itself. The appeared/disappeared interfacial free-volume voids are not too large, since the characteristic value of *τ*_int_ = 0.27 to 0.30 ns lifetime can be associated with typical volumes of mono- or diatomic vacancies in chalcogenide-like systems [1,3,14-16].

It is also worthwhile to mention that free-volume interfacial voids in glassy samples affected by more essential fragmentation (50-h annealed samples taken in respect to 10-h annealed ones) are larger as compared with agglomerated free-volume voids (the corresponding value of defect-related *τ*_int_ lifetime reaches 0.370 ns). This finding occurs to be in good agreement with a known trend towards dimensional enlargement of such voids occurring under decrease in the nanoparticle sizes [17].

## Conclusions

Positronics of atomistic imperfections such as free-volume interfacial voids in nanostructurized solids with positron-trapping modes changed by incorporated nanoparticles is developed as unified mathematical algorithm of substitutional positron-positronium trapping in the same host matrix. Within developed formalism, grounded on coupling x3-x2-decomposition procedure, the physical characteristics of nanostructurized media can be well calculated to estimate (1) the defect-related positron lifetime linked to interfacial void volumes responsible for positron trapping and (2) the defect-free bulk positron lifetime of the agglomerated nanoparticles.
